# Clustering analysis of factors affecting academic career of university students with dyslexia in Italy

**DOI:** 10.1038/s41598-022-12985-w

**Published:** 2022-05-30

**Authors:** Ilaria Benedetti, Marcella Barone, Valentina Panetti, Juri Taborri, Tony Urbani, Andrea Zingoni, Giuseppe Calabrò

**Affiliations:** University of Tuscia, 01100 Viterbo, Italy

**Keywords:** Neurological disorders, Learning and memory

## Abstract

This study was designed to explore learning experiences of university students with dyslexia and factors that could contribute to their success in the university career. Although, great efforts have been made to diagnose dyslexia and to mitigate its effects at primary and secondary school, little has been done at the university level in particular in the Italian context. Indeed in the university context, the availability and possibility to use of support tools, that enable the student to achieve educational success, is still not sufficiently adequate. In this paper we used bivariate association tests and cluster analysis, in order to identify the most suitable compensatory tools and support strategies that can facilitate the students’ performance in higher education. The data were obtained through the voluntary participation of Italian students, enrolled in a bachelor degree course, with certified diagnosis of dyslexia. Six groups of students were identified from the cluster analysis, defining specific support tools and learning strategies for each group. Furthermore, through the creation of these six groups, it was possible to describe “profiles” that highlight the risk factors (late diagnosis) and-or protection factors (such as associations, support from friends and family) in analyzing the academic career of students with dyslexia. Therefore, starting from these data, through artificial intelligence it will be possible to identify and suggest study methodologies and create specific support tools for each student that can enable her/him to achieve educational success in her/his academic career.

## Introduction

Dyslexia is a specific learning disorder (SLD), in that makes affected people continue to experience significant reading and writing related problems throughout their adult lives, which has been generally explained in terms of phonological deficits^1^. Adults with dyslexia experience significant difficulty in adjusting to the academic demands of higher education^2^. Indeed, students with dyslexia often experience problems with information processing, note-taking, organization of essays and expressing ideas in writing^3,4^. Dyslexics students try to compensate for reading and writing disorders by using a variety of compensatory strategies including memory aids, advocacy strategies and digital tools^5^. Moreover, a lack of confidence can affect students’ performance, especially in social situations, such as reading and writing in front of others. Thus, enhancing learning potential of students with dyslexia is still an open challenge for academic staff^6^. Internationally, the number of students with disabilities enrolled in higher education is on the rise^7^. Students with dyslexia compile the largest subgroup^8^. In Italy, a survey conducted by ANVUR (National Agency for the Evaluation of the University System and Research) together with CNUDD (National University Conference of Rectors’ Delegates for Disabilities and Dsa) during 2020, reveals that between 2017 and 2020, students with SLD increased from 6500 to 16,084.

Starting from Goal 4 of the Sustainable Development Goals, which calls for equal access to tertiary education, including university, as part of the promotion of lifelong learning opportunities for all and in particular for the vulnerable people, the aim of this paper is to identify a set of approaches to deal with the difficulties encountered by dyslexic students in terms of homogeneity in socio-demographic characteristics, the difficulties encountered during university experience with the ultimate goal to provide appropriate treatment and support needed for successful functioning in and out of school. In this paper we used bivariate association tests and cluster analysis, which is a multivariate statistical analysis tool, in order to understand the most suitable compensatory tools and support strategies that can facilitate the students’ performance. An insight into their lives and experiences with an overview of their past is evaluated. The sample includes students that are studying or complete the career less than five years agos, in Italian universities, plus students enrolled in the last 2 years of High School. Data has been collected by using a self-reported questionnaire method. In our study we only considered certified dyslexic students, for whom the clinical diagnosis of dyslexia is based on the elements that foresee and comply with the recommendations of the Consensus Conference (2007–2009 and subsequent updates) and the Consensus Conference of the ISS (Superior Health Institute). The volunteers of this study were undergraduate and postgraduate dyslexic students studying for a degree in Higher Education. The study is within an European project, named as VRAIlexia, that aims at providing tools and services for supporting the academic life of dyslexic students. More specifically, the tangible outcomes of the project can be summarized as follows: (1) a battery of virtual reality test useful to both collect in real-time the skills of dyslexic students through quantitative scores and enhance the awareness of teachers; (2) an artificial intelligence-based platform for the identification of the most appropriate supporting methodologies based on student-specific needs, whose proof of concept is presented in^9^; (3) realize of an online shared repository of learning and teaching materials; (4) create training paths for both students, based on the enhancement of the entrepreneurial mindset, and teachers, for improving the dyslexia awareness and (5) a sketching a memorandum of understanding to spread common inclusion strategies among higher education institutions. The remainder of the paper is structured as follows: “Background and literature review: higher education and dyslexia in the Italian context” reports the background of SLD and literature review in the italian higher education context, in “Data” we describe the survey and the acquired data, whereas in  “Method” we report the used method for the analysis. Finally, in “Results” we discuss the main results and in “Conclusion” we conclude.

## Background and literature review: higher education and dyslexia in the Italian context

Since Dyslexia, as SLD, tends to persist throughout life and constitute a potential factor of vulnerability, there is a recognition of the need for a range of support over time and the importance of early diagnosis. The assessment of SLD must be a process guided by criteria as well-defined as possible, according to standards contained in the diagnostic manuals. Over the years, a lot has been done to diagnose dyslexia. Diagnosis has usually relied on some specific tests that aim to quantify reading difficulties, jointly with clinical tools that measure cognitive abilities^9^. Despite these fundamental guidelines that emerged, the Italian community of clinicians highlighted that there are some critical aspects and started a debate on the matter as early as 2014 “the criteria adopted in general for the diagnosis of SLD must be respected, such as the absence of a decisive role of environmental and educational factors, and other exclusion factors such as sensory and intellectual handicap and severe emotional problems”^10^. In recent years the debate around the issues concerning the modality of the diagnosis of dyslexia has been further animated also at the international level. One of the most debated issues is the IQ-achievement discrepancy model that it does not assess or inform the quality of instruction received by students. Some students may be identified as having learning disabilities when, in reality, they simply have not experienced classroom instruction that meets their learning needs. A new approach to the diagnosis of dyslexia has been proposed by^11^. Otherwise as concluded by^12^, it’s very important that clinicians, schools and researchers cooperate in developing tools with good psychometric properties and based on recent research on dyslexia. In Italy, Law no. 170/2010 (New Rules on Specific Learning Disorders in schools) recognizes dyslexia, dysgraphia, dysorthography and dyscalculia as as SLD. The 170/2010 law has the purpose of: (a) guarantee the right to education; foster academic success, (b) including through educational support measures, ensure adequate training and promote the development of potential; (c) reduce relational and emotional discomfort; (d) adopt forms of verification and evaluation adequate to the training needs of students; (e) prepare teachers and raise parents’ awareness of problems related to learning difficulties; (f) to favor early diagnosis and rehabilitation didactic paths; (g) to increase communication and collaboration between family, school and health services during the education and training process; (h) ensure equal opportunities for the development of social and professional skills. The operating profile ascertains: cognitive skills; Linguistic and metaphonological skills; Visual-spatial skills; Motor-praxic skills; Attentional skills; Memory skills; School skills: reading, writing (spelling, written expression, handwriting), reading comprehension, calculation, study method; Affective-relational situation (self-esteem, motivation, relational skills with peers and adults). The diagnostic evaluation can only take place in the structures of the national health system or those accredited by it. It has a multidisciplinary character, in fact, the team is made up of a child neuropsychiatrist, a psychologist and a speech therapist or psychomotor specialist. The clinical diagnosis is based on the elements that foresee and comply with the recommendations of the Consensus Conference (2007–2009 and subsequent updates) and the Consensus Conference of the ISS (Superior Health Institute). In the diagnosis, explicit reference is made to the nosographic criterion as indicated by the ICD-10: F81, also specifying whether reading and-or calculation and-or writing disorder is thus configured as a mixed disorder. The certification contains the information necessary to draw up an individualized and personalized educational and didactic program, where the dispensatory measures and the compensatory tools useful to the student for his educational path are explained. In fact, an operating profile is formulated that highlights the student’s weaknesses and strengths. The certification is always updated through a reassessment at the authorized structures at each change of school cycle and not earlier than 3 years from the previous one. Or upon notification by the school or family where an update is necessary. Specifically, Law no.170/2010 set methods, including medical history, clinical interview, school report, teacher evaluation, rating scales, and psychometric assessment to be considered for the dyslexia evaluation^13^. Finally, it is important to specify that in Italy there are no schools or special classes for students with dyslexia, but that an inclusive path is favored thanks to the law 170/2010 that provides dyslexic students with the necessary tools to achieve educational success. Law 170/2010 was the first specific regulatory intervention in Italy on SLD which ensures that students with SLD can benefit from special compensatory measures of didactic flexibility during the course of education and training and in university studies. Moreover, it requires that school institutions guarantee “the introduction of compensatory tools, including alternative means of learning and information technologies, as well as dispensing measures from some non-essential services for purposes of the quality of the concepts to be learned”. Compensatory tools are devices that raise the student from the performance made hard by the disorder, allowing him/her to focus on more complex cognitive tasks. On the other hand, dispensary measures are interventions that allow the pupil or student not to perform certain services that, due to the disorder, are particularly difficult and that do not improve learning. Law 170 provides that the assessment of dyslexia takes into account neuropsychological, psychological and speech therapy aspects. After the diagnosis evaluation, the family role is of the utmost importance l since parents are involved in the establishment of an educational plan with the school, by defining their rights and duties, as well as providing the authorization to apply suitable compensatory instruments and dispensatory strategies^14^. Moreover, family support can be a protective factor that may positively impact self-esteem^15^. Confirmation of the diagnosis is required at every change in school level and whenever it is deemed useful to modify it, to take into account the possible fluctuations of the problems and possible significant recoveries^10^.

Dyslexia is one of the most common conditions experienced by school-age children^16^. It is worth noting that in Italy dyslexia is not recognized in almost two out of three children at the age of 8–10 years, when the disorder should be clearly expressed and identified. The non-recognition of two-thirds (or more) of the cases of dyslexia and the lack of adequate and timely interventions lead to anxiety and depressive behaviors^17^, low self-esteem and a low academic self-concept with school failure and drop out^18^. Focusing on high school students, academic assignments can have an impact on self-esteem and self-confidence, particularly when written assignments attract criticism for their poor presentation and weaknesses of grammar, punctuation and spelling^19^. Moreover, children with specific learning disorders are often subject to stigmatization by families, teachers and peers, which can lead to increased self-stigma and reduced motivation to learn^20^.

To the author’s knowledge, only few studies have focused on identifying emotions among dyslexic students. Among them^21–23^, and^24^ found that students with dyslexia experience high levels of stress, strong negative emotions such as fear and loneliness, due to their interactions with teachers. Other dyslexic students report feelings of lack of confidence, inferiority^25^ and anxiety^26^. Moreover, other studies reported that students with dyslexia have a significantly lower level of self-esteem and more negative self-concept when compared to other students^27,28^.

It is worth noting that facing the diagnosis of dyslexia can be emotionally and psychologically challenging: recognizing it early, especially before the beginning of school, is crucial to help affected people fill their learning gap^29^. Indeed, the earlier the intervention, the better the expectations of effectiveness. The management of SLD requires rehabilitative and educational care. The rehabilitation process is carried out as early as possible. Effectiveness is linked to the precociousness, intensity and frequency of interventions, for which the rehabilitation can rely on the collaboration of the family and the school. The earlier the intervention, the more likely it is that the procedures to help children with difficulties meet the normal teaching procedures of the class. A study carried out in Italy, revealed that the late diagnosis of dyslexia (in adulthood) is associated with negative effects, such as a sense of shame and incompetence, whereas in the case of early diagnosis or when parents and/or friends supported them adequately, this enabled students to cope with their dyslexia^30^. Another factor that negatively affects the academic lives of students with dyslexia is the number and severity of difficulties encountered^31^.

University students with dyslexia have been inadequately investigated to date, especially in Italy^27^. A few previous studies have nonetheless demonstrated that this permanent disorder can cause undergraduates and other adults a number of difficulties when they have to cope with tasks and activities that involve reading and writing^32,33^.

The biggest problem, in Italy, is the inadequacy of diagnosis tools for post-school age. Indeed, when students with a late diagnosis arrive at university, these difficulties are overlooked and it is no longer possible to see improvements in the disorder but only refinement of the study method with the aim to compensate for the problems, thus making explicit the importance of identifying the appropriate support methods. To date, very few studies have explored day-to-day learning experiences of university students with dyslexia. For this reason, the aim of this work is to analyze a representative sample of the Italian population of students affected by dyslexia in order to identify the socio-demographic characteristics, the perceived difficulties and supports needs useful for each “type” of diagnosed-dyslexic student entering the university pathway ho have been diagnosed with dyslexia.

## Data

**Figure 1:**
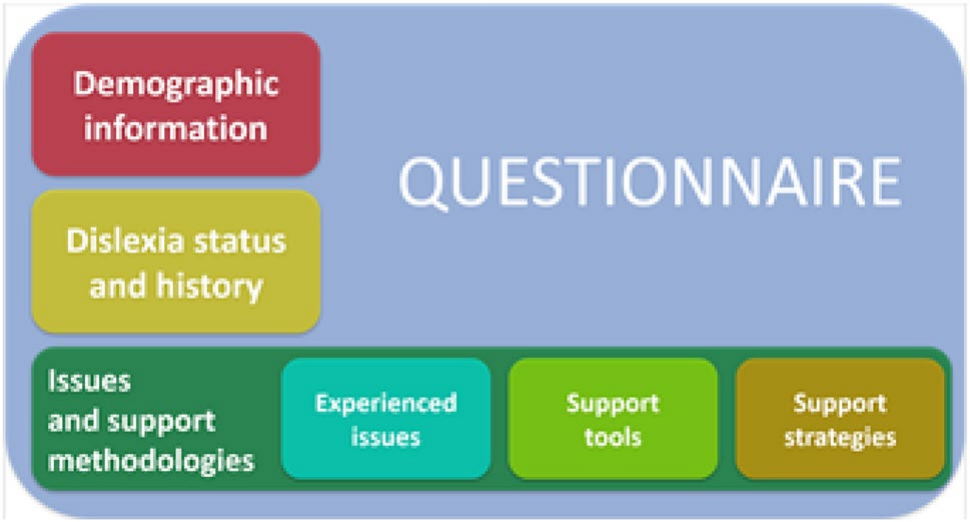
Scheme of the questionnaire administered to dyslexic students.

The collection of the data needed to achieve the goal of the study was carried out through a questionnaire that was administered to Italian students with dyslexia, who were at least 18 years old and who were attending university or left it at most 5 years earlier. Our questionnaire was subjected to a double conformity check. Indeed, after an internal validation of the questionnaire by the ethics committee of the University of Tuscia, we sent our research proposal to the National University Conference of Disability Delegates (CNUD)—which is a body to represent the policy and activities of Italian universities towards students with SLD and disability. The attached questionnaire was validated in accordance with CNUD’s ethical principles and guidelines. The Italian Universities that have taken part in the survey are only those that have responded favorably to the CNUD’s invitation, thus ensuring that the ethical principles of their respective Universities are respected. In total, 66 Italian universities took part in the survey. Moreover, the data collection was conducted according to the ethical standards outlined in the 1964 Declaration of Helsinki. After that the Italian Universities have taken part in the survey, the students who completed the questionnaire were recruited by the Rector’s delegates for Inclusion at each University on the basis of the certification submitted at the time of enrolment at the university. The certificate is accepted by the University if the clinical diagnosis is based on the elements that foresee and comply with the recommendations of the Consensus Conference (2007–2009 and subsequent updates) and the Consensus Conference of the ISS (superior health institute). If a student has been diagnosed with a specific learning disorder (SLD) and present a certificate (drawn up in accordance with the terms of Italian law no. 170/2010 and issued not later than 3 years ago by the Italian Health Care System (SSN) or by a specialist or by an health care facility recognized by the Italian system) the Special Needs Unit will make sure that, once he/she is enrolled at University, get the necessary arrangements in order to: facilitate his/her academic progress and success; ensure a proper educational path;—develop his/her potential; reduce the causes of educational and emotional discomfort. The following services are offered by the Universities to the SLD students: support for prospective students who want to take the admission test; tutoring service to provide information and advice, promote integration at the university and suggest a method of study for core subjects; opportunities for interaction with the teaching staff in order to identify the best ways to attend courses and take exams. The participation in the questionnaire was voluntary and all the data has been recorded, collected and analyzed by guaranteeing anonymity and the respect of the General Data Protection Regulation guidelines. All the participants were informed about the aims of the study and asked to digitally sign an informed consent. The questionnaire is basically divided into three sections as sketched in Fig. 1.

The first one comprises demographic questions, such as age, gender, attended degree course and/or school and year of the program. The second section investigates the dyslexia status and history of the students. It includes questions about co-morbidity with other SLDs, type of support received, year of the diagnosis and presence of relatives with dyslexia. For each question, some answers are suggested. Suggestions are based on the corpus of knowledge about dyslexia and cover quite exhaustively all the possible answers. However, a blank space where to add possible missing alternatives has been inserted, in order not to neglect any useful information and not to bias the test. The third section, instead, is focused on the issues experienced by the students during their learning process and on the support tools and strategies that they found particularly useful or useless. This section is in turn divided into three groups, which survey issues, support tools and support strategies, respectively. For each group, a long list of items is presented. The participants must give a score from 0 to 5 (considering only integer values) to each item, where 0 stands for not experienced issue or useless tool/strategy, 5 stands for very strong issue or very useful tool/strategy and the marks in the middle stand for intermediate levels of importance of the issue or usefulness of the tool/strategy. The options “unknown” and “never tried” have been added in the support tools group, in order to differentiate such cases from the 0 mark, which means “tried and considered useless”, instead. The three lists of items have been created from face-to-face interviews made to 25 dyslexic students, by including all the issues they experienced, also if partially or just for a brief period of their life, and all the support tools/strategies they tried or they would like to have tried. This allowed having an exhaustive overview. However, again, a blank space has been left to give the opportunity to the participants to indicate possible missing issues/tools/strategies. It is worth noting that completing the questionnaire takes less than 10 min. In fact, it was specifically designed to be light and not too time-consuming, since performing large and repetitive tasks is one of the problems that most severely affects people with dyslexia. This allows participants to be more engaged, so as to provide answers unbiased by lack of concentration or hurry and, thus, more reliable data. We adopted a stratified probabilistic sample. The primary sampling units are represented by universities, while the secondary sampling units are students. We considered a sample with a sampling fraction equal to 10% of the total population. In this way we obtained a sample population that best represents the entire population being studied. The questionnaire was hosted online and 1.261 students with dyslexia from across Italy answered it. 72 answers were discarded since the participants were either outside the age range, or did not have a dyslexia clinical report, or completed the questionnaire more than once. The remaining 1.189 answers constitute the database of the analysis performed in this study.

## Method

We here adopted an agglomerative algorithm in order to apply a cluster analysis, which is a multivariate statistical tool aimed at grouping a set of units in such a way that units in the same group (called as a cluster) are more similar to each other than to those in other groups (clusters). This method is the most common type of hierarchical clustering used to group units in clusters based on their similarity since it is suitable for constructing and identifying typological groupings that can be both distinct from each other and internally homogeneous^34^. In our study, the units of analysis were students who participated in the survey. Agglomerative hierarchical clustering techniques proceed by a series of successive mergers. It starts with the individual units, thus there are initially as many clusters as units. The most similar units are first grouped, and these initial groups are merged according to their similarities. Eventually, as the similarities decrease, all subgroups are fused into a single cluster. Concerning the measure of similarity, we used the Gower distance^35^. The Gower distance is a metric that measures the dissimilarity of two items with mixed numeric and non-numeric data. Gower distance is also called Gower dissimilarity. This is the most popular distance for mixed-type variables; it is appealing because ranges between 0 and 1, being an average of the scaled distances calculated variable by variable. The Gower’s distance can be defined as the complement to one of the Gower’s similarity coefficient:

$$d_{G,ij}=1-s_{G,ij}=\frac{\sum _{t=1}^{p}\delta _{ijt}d_{ijt}}{\sum _{t=1}^{p}\delta _{ijt}}.$$

It is a dissimilarity or distance measure^36^ between unit i and unit j, where $d_{ijt}=1-s_{ijt}$ is the distance calculated on the t-th variable, $s_{ijt}$ is the similarity between i and j with respect to the t-th variable and its value depends on the type of the variable itself. After the definition of the distance measure, it is necessary to concentrate on the linkage methods: single linkage (minimum distance or nearest neighbor), complete distance (maximum distance or farthest neighbor), average linkage (average distance) and Ward linkage. Ward^34^ is considered hierarchical clustering procedures based on minimizing the “loss of information” from joining two groups. This method is usually implemented with loss of information taken to be an increase in an error sum of squares (ESS) criterion. Firstly for a given cluster k, let ${ESS}_k$ be the sum of the squared deviation of every item in the cluster from the cluster mean (centroid). If there are K clusters, define ESS as the sum of the ${ESS}_k\ or\ ESS={ESS}_1+{ESS}_2+\cdots +{ESS}_k$. At each step, the union of every possible pair of clusters is considered, and the two clusters whose combination results in the smallest increase of ESS (minimum loss of information) are joined. Initially, each cluster consists of a single item, and, if there are N items, ${ESS}_k=0,\ k=1,2,\ldots ,N$ then ${ESS}=0$. At the other extreme, when all clusters are combined in a single group of N items, the value of the ESS is given by: $ESS=\sum _{j=1}^{N}\left( x_j-\bar{x}\right) \prime \left( x_j-\bar{x}\right)$ Where $x_j$ is the multivariate measurement associated with the j-th item and $\bar{x}$ is the mean of all the items. The results of the agglomerative method may be displayed in the two-dimensional diagram known as dendrogram. The dendrogram illustrates the merges that have been made at successive levels.

## Results

### Results from descriptive statistics

This analysis considers a representative sample of 1.189 adulthood students with dyslexia. Dyslexia represents the most frequent self-declared disorder in higher education. Participants varied in terms of age, gender, level of study and age of diagnosis reflecting diversity within the students population. We start our analysis by considering some socio-demographic aspects of the sample. Table 1 reports frequency distributions for the main sample’s characteristics.

**Table 1:** Socio-demographic characteristics of the sample.

Gender		Diagnosis of dyslexia	
Male	66%	Primary school	41%
Female	24%	Secondary school	20%
Type of student		Tertiary school (1st or 2nd year)	13%
Full-time student	83%	Tertiary school (3rd–5th year)	25%
Part-time student	17%	Year of birth	
Received aid		< 1990	3%
No	34%	1990–1999	52%
Yes	67%	> 1999	45%
Type of aid received		Year attended	
Private speech therapists	23%	First year	36%
Psychologist	18%	Second year	24%
Public speech therapists	16%	Third year	18%
Tutor	13%	Fourth year	4%
Parents	3%	Fifth year	3%
Teacher	1%	Out-of-study student	10%
Dyslexia Association and friends	2%	Graduate student	3%
Other learning disorders		High school student	2%
No	21%	Relatives with dyslexia	
Yes	79%	No	57%
		Yes	43%

**Figure 2:**
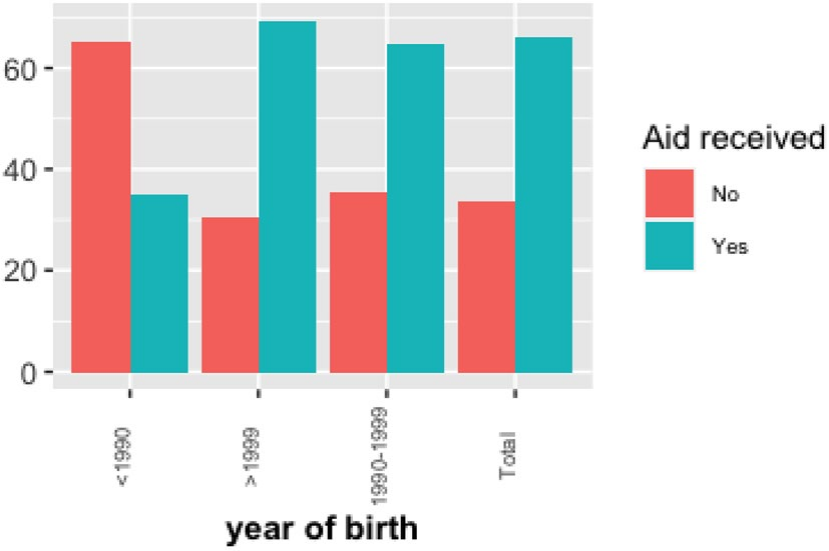
Student’s age and received support.

From Fig. 2, we can see the association between the student’s year of birth and whether or not they received any form of support after the diagnosis (Pearson chi2(2) = 13.3354 Pr = 0.001). From the value of the Chi2 and the p value we can support that a strong association exists between the two variables. Indeed, as reported in “Background and literature review: higher education and dyslexia in the Italian context”, In Italy the first law recognizing dyslexia, , dysorthography, dysgraphia, and dyscalculia as specific learning disorders was enacted in 2010, Law 170/2010 protects the right to study of dyslexic children and gives the school an opportunity to reflect on the methodologies to be put in place to benefit all students, giving space to their true potential according to their peculiarities.

Indeed, Fig. 2 shows that among students born before 1990, only 35% of them received support (compared to 67% of the total sample). Therefore, the introduction of Law n.170 has produced effects on the possibility of receiving aid.

**Figure 3:**
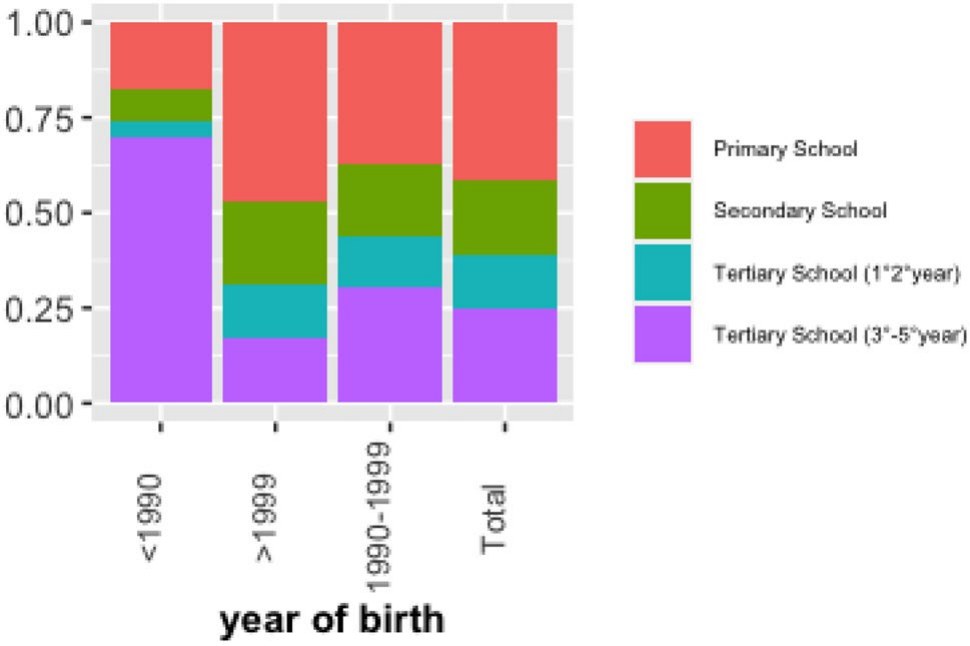
Time diagnosis and student’s year of birth.

From this consideration, in Fig. 3 it is interesting to note that 70% of students born before 1990 received dyslexia diagnosis during tertiary school (3rd–5th year), thus revealing a strong and significant relationship between year of birth and when the student received the diagnosis (Pearson chi2(3) = 53.03 Pr = 0.000). Indeed, the percentage of students born after 1999 who received diagnosis during tertiary school (3rd–5th year) is equal to 17%. Moreover, this association underlines the growing awareness in SLD since 2010 for an inclusive education model.

**Figure 4:**
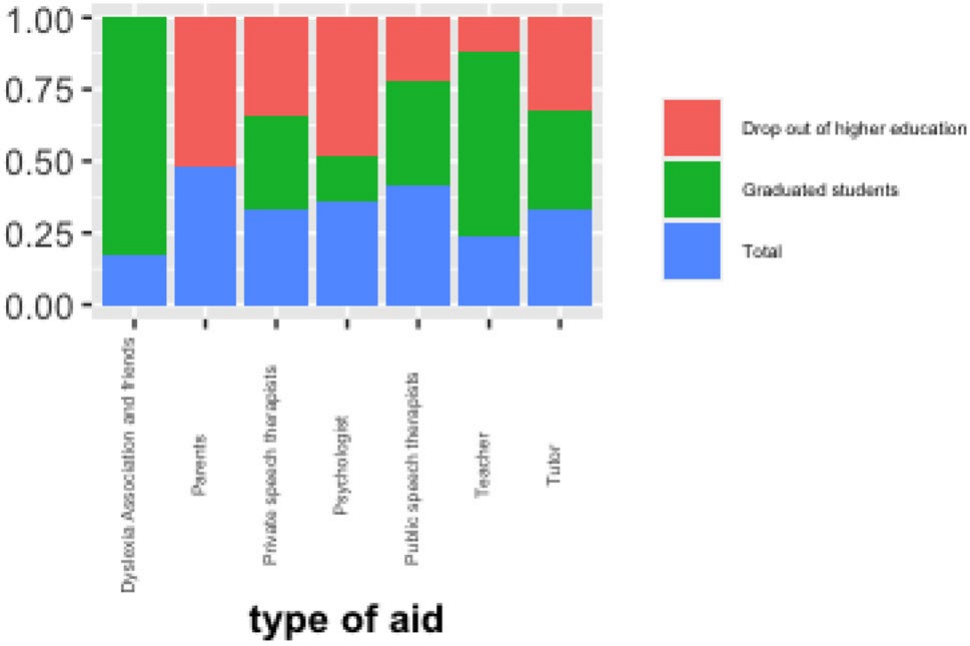
type of support used by type of student.

By considering the type of support in each student’s type, significant relationships emerge from Fig. 4. In particular, among graduate students, there is a high percentage of students who have received support from associations and friends. On average, help from associations and friends is chosen by 80% of graduate students (Pearson chi2(3) = 13.22 Pr = 0.006), whereas among students who dropout university education, it emerges a strong association with supports from parents (Pearson chi2(3): 14.54 p value 0.042). Indeed, students are more successful when early in their lives someone has been extremely supportive and encouraging, and when they have found an area in which they can succeed. From these results it can be concluded that it is important to encourage teamwork studying. Small group helps many students with dyslexia. People have strengths in different areas; working with a group increases the chances of problem solving. Working on group projects can take advantage of each individual’s strengths; some are good at writing, others at drawing, others at research, and others at building models. Indeed, our results confirm the positive role of social capital in health at the level of macro-organizations, national associations, essentially through three processes^37^: better access to relevant information, lobbying activity and access to a self-care network. Belonging to an association that deals with dyslexia guarantees students access to a network of opportunities and relationships that also have a significant impact on the student’s self-esteem. A recent research on dyslexic university students^38^ confirms the need of students in helping to create a community of support and support for dyslexic students within universities. Even at the level of micro-groups, social capital has a powerful balancing effect, both in terms of results and the self-esteem of students.

### Results from cluster analysis

**Figure 5:**
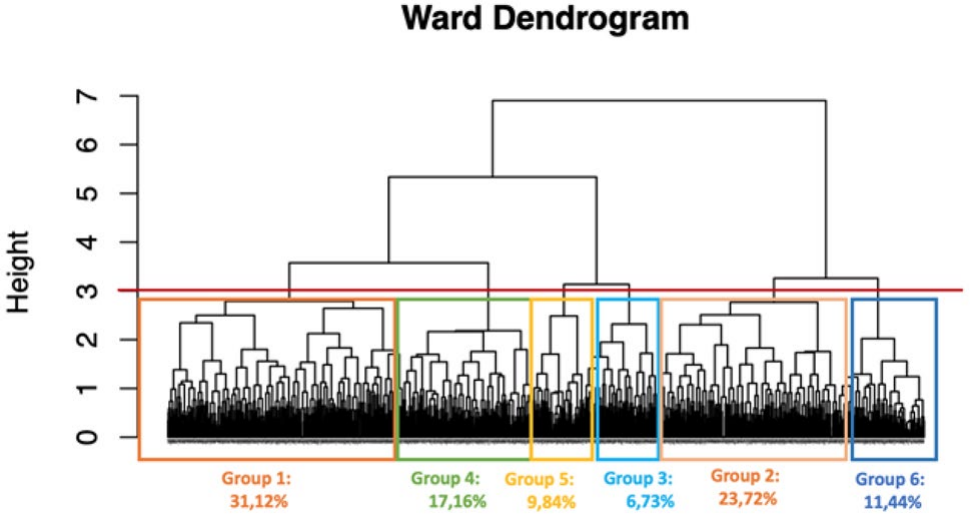
Cluster dendrogram.

In this section, we report the results from the cluster analysis. As shown earlier, we used a hierarchical agglomerative method via Gower’s distance measure. We relied on agglomerative coefficient analysis to choose the link type. Indeed, this indicator measures the dissimilarity of a unit with respect to the first cluster to which it joins, divided by the dissimilarity of the final merger in the cluster analysis, averaged over all samples, we chose the best criterion among those presented above. Low values of the agglomerative coefficient reflect clustering of units that are “heterogeneous” with each other, larger values indicate well-formed clusters with “homogeneous” units in each cluster. From the analysis of the agglomerative coefficient, we chose to use Ward’s method, a hierarchical method that is based on the decomposition of total deviance into deviance within groups and deviance between groups; at each interaction the pair that gives rise to the lowest variance within groups is merged. The process of aggregation can be represented by using the dendrogram, which reports on the x-axis the units that participate in the process of aggregation and in the y-axis the level of distance at which the aggregation occurs between different groups that are being formed for successive agglomerations. From Fig. 5, it is possible to obtain useful indications regarding the number of groups to be considered, in this case equal to six (in brackets the dimension of each cluster). In correspondence of the levels in which the distance (height included in the y axis) between the groups grows clearly, it means that the aggregation happens at an elevated cost and it is therefore convenient to stop the process.

In order to proceed with the clustering process, we considered in the analysis the issue that students have encountered during their university career and the socio-demographic characteristics, based on the questionnaire answers. In the following table we report the six groups identified by the cluster analysis according to year of birth.

**Table 2:** Groups identification by considering socio-demographic characteristics.

	Group 6—at-risk students	Group 3—graduated students
Year of birth	Before 1990	Before 1990
Type of student	Out-of-study students or dropout students	5th year of university (grad students) or Graduated student
Diagnosis of dyslexia	3–4–5 years of High school	Primary school
School year repetition	Yes	No
Type of aid received	No	Parents
Relatives with dyslexia	Yes	No
Other difficulties	Yes (all)	Yes (all)

	Group 2—at-risk students	Group 5—graduated students
Year of birth	1990–1999	1990–1999
Type of student	4th or 5th year of university, out-of-study student	3rd year of university (grad students), Graduated student
Diagnosis of dyslexia	3–4–5 years of High school	1–2 year high school
School year repetition	Yes	No
Type of aid received	Parents and Tutor	Dyslexic association and friends
Relatives with dyslexia	Yes	No
Other difficulties	Yes (dyscalculia)	No

	Group 1—at-risk students	Group 4—students of the future
Year of birth	After 1999	After 1999
Type of student	1st–2nd years of university, 4th or 5th year of university (part-time students)	High school students and 1st–2nd years of university
Diagnosis of dyslexia	Primary–secondary school	1–2 year high school
School year repetition	Yes	No
Type of aid received	Public and private speech therapists, teacher	Private speech therapists and psychologist
Relatives with dyslexia	No	No
Other difficulties	Yes (dysgraphia)	Yes (dysgraphia-dysorthography)

As we can see from Table 2, an early and effective diagnosis is a key aspect of the student’s success in university studies. It should be desirable to get an earlier diagnosis (during the first years of primary school) in order to activate early rehabilitation activities that could help contain dyslexia. Dyslexia can be contrasted more effectively with early and specific interventions. Students who do not get support until later grades may have more learning difficulties, in particular in the skills needed to read well. They are likely to lag behind academically and may never be able to catch up. A child with severe dyslexia may never have an easy time reading, but he or she can learn skills that improve reading and develop strategies to improve school performance and quality of life. Group 3 and group 6 includes mainly students born before 1990, they started their university studies before the introduction of law 170/2010 which recognizes dyslexia, dysorthography, dysgraphia, dyscalculia as SLD in Italy. Although the two groups are characterized by not having received aids, the students who fall into group 3 are able to conclude their university studies, whereas those who fall into group 6 are mainly students who have not passed all their exams within the prescribed period of time and tend to abandon their studies. As we will see from Table 3, this difference in the success of the course of study can be connected to the intensity of the difficulties encountered by the students included in the two groups. Group 3 is characterized by including students with a low level of disorder severity, whereas group 6 is characterized by including students with many widespread difficulties. Groups 2 and 5 mainly include students born between 1990 and 1999. Group 5 represents 10% of the sample and could be called the “success” group, since this group includes students who have already concluded their university studies. They were born between 1990 and 1999 and although they received the diagnosis of dyslexia “late” (first-second year of university) they received specific help from associations, friends and psychologists. Group 2 represents 24% of the sample and it includes students born between 1990 and 1999. Students in this group are currently attending 4th or 5th year of university or enrolled in supplementary years. They received a diagnosis of SLD in the last 3 years of high school and received support from tutors and parents. Groups 1 and 4 include mainly students born after 1999. Group 1 represents 31% of the sample and includes children born after 1999 and currently attending university as part-time students. They received a diagnosis of SLD during elementary and secondary school and they received specific help from public Speech Therapist, Private Speech Therapist and Teacher. Group 4 (similar to group 1) represents 17% of the sample and it includes children born after 1999 and currently attending high school or the first 2 years of university, they could be named as the group of “students of the future”. They received a diagnosis of SLD during secondary school and the first 2 years of high school (therefore slightly later than group 1) and were supported by a private speech therapist and psychologist. They had been diagnosed with dyslexia during their secondary school years by specialist services forming part of the Italian National Health System. The last section of the questionnaire asked participants about their support needs. This theoretical viewpoint of learning and study strategies is concerned with the cognitive strategies that students apply to learning contexts. As shown in Table 3, the difficulties most frequently endorsed by dyslexic students were concentration and remembering facts, indeed they need supplementary tools in note taking, organizing the lessons and exams. As we can see from Table 3, two forms of learning strategies were reported by more than half of students: group activities and exam assessment. Among group activities the most commonly reported strategies are: fellow student ‘study buddies’, support groups, study skills or literacy, support workshops and to be part of dyslexic students association, while among the exam assessment, the most commonly reported strategies are: oral examination, exam with tenured professor and the possibility to divide the exam. The remaining strategies that were most commonly reported are: provision of lecture notes and overheads, taped lectures provided, face-to-face classes. Moreover, as reported by^39^ in order to increase reading accessibility for those who struggle the most, text simplification might be used as an efficient rehabilitation tool and daily reading assistive technology, fostering overall reading ability and fluency through increased practice.

**Table 3:** Difficulties, support tools and learning strategies by groups.

	Difficulties	Support tools	Learning strategies
Group 6	Many widespread difficulties	Coloured overlays, easy-reading font, tutorial support	Group activities, Lecture support, exam assessment
Group 3	Few difficulties on Concentration and remembering facts	Audiobook, voice activated technology	Oral exam
Group 2	Concentration and remembering facts	Audiobook, voice activated technology, smart-pen e tablet, clearer layout, e-book, pictures to understand meaning	Group activities, exam assessment
Group 5	On-line lessons, exam organization	Audiobook	Group activities, exam assessment (oral exam)
Group 1	Concentration, on-line lessons, remembering facts	Use ready-made maps, diagrams and summaries, smart-pen e tablet, clearer layout, easy-reading, pictures to understand meaning	Group activities, lecture support (taped lectures provided, lecture notes provided)
Group 4	Concentration, remembering facts, exam organization, handwriting, expressing ideas orally and writing	Audiobook, clearer layout, easy-reading, keywords, use ready-made maps diagrams and summaries, e-book, tutorial support, pictures to understand meaning, video lessons, internet searches	Group activities, exam assessment (oral exam)

Participation into study groups may have a positive effect on the well-being of dyslexics students, this participation could enable favorable relations, reciprocal acceptance, and pleasant involvement in group activities. On the other hand, it is important to remember the importance of social partner interventions aimed at improving self-image, greater development of communication skills and increased motivation to learn^40^. In addition, interactive discussions and classroom activities can increase self-esteem in students with SLD^41^.

## Conclusion

The number of dyslexic students in need of support is increasing at many universities^7^. University students with dyslexia have disparate and complex needs that may require a mix of supporting tools. Students with SLD experience feelings of failure within the school education system, in particular if their problem is not recognized and adequate support is not provided. Providing appropriate support can fulfill the reading and writing requirements in higher education. The aim of this study was to explore the lives and experiences of dyslexic students studying at Italian Universities in order to provide appropriate support tools and strategies that can facilitate the students’ performance. The research took place between March and May 2021 by collecting 1.189 questionnaires. In this paper we used bivariate association tests and cluster analysis, which is a multivariate statistical analysis tool. Our results from descriptive statistics reveal the importance of an earlier diagnosis and treatment for dyslexia with specific help, in particular help from association of dyslexic students. Earlier diagnosis, treatment for dyslexia with specific help from speech language therapists, psychologists and association and being able to compensate for dyslexia, by providing appropriate equipment can be keys to success in university studies in order to help dyslexic students to feel in control of their learning. In this context, it is important to note that from our results emerges a positive association between help from associations and friends and the probability of success in university studies. Belonging to an association that deals with dyslexia guarantees students access to a network of opportunities and relationships that also have a significant impact on the student’s self-esteem. A recent research on dyslexic university students^38^ confirms the need of students in helping to create a community of support and support for dyslexic students within universities. Even at the level of micro-groups, social capital has a powerful balancing effect, both in terms of results and the self-esteem of girls and guys. Moreover, our data, international scientific literature and recent research at national level confirm that it would be essential to support and implement macro, meso and micro support networks for dyslexic students with ad hoc policies and strategies, formal or informal. Finally, results from cluster analysis suggest a variety of internal challenges associated with attending higher educational institutions: universities are to actively promote students’ equal rights and prevent direct or indirect discrimination and they should guarantee individualized and personalized didactic plan. In Italy, awareness of SLD issues is very recent, as the first law recognizing dyslexia was introduced in 2010. For this reason it is necessary an extra effort on the part of universities to make their institutions more dyslexia-friendly across the board. This can include everything from using dyslexia-friendly fonts, stricter guidelines for teaching staff regarding monitoring student action plans and social skills interventions should be a popular adjunct treatment for students with SLD. In the light of the results obtained from the survey carried out over 1.189 certified Italian dyslexic students, this study intended to identify, through the statistical cluster analysis, which tools may be beneficial to the development and adaptation of educational methods to support students with dyslexia in order to overcome all the main difficulties encountered. Indeed, as already reported in^9^ dyslexic university students often see their academic career slowed down or even spoiled. Starting from the necessity to untap dyslexic students’ potential and enhance their strengths, the results of this research will be used to develop learning tools and services to ensure to dyslexic students equal access and opportunity of success during their career and their lifelong learning experience. The results of this research will be part of the project VRAIlexia (virtual reality and artificial intelligence for dyslexia), i.e. the output of the cluster analysis survey-based proposed will be part of BESPECIAL AI (Artificial Intelligence)-based platform. Indeed, the AI solution we propose is a software platform able to provide dyslexic students at university with customized digital supporting tools and personal learning strategies. The kernel of the platform consists in an AI-based module that is capable of predicting the specific support needed by each user, from his/her dyslexia clinical report and, the answers to a self-evaluation questionnaire about the difficulties faced during the studies and the solutions they deem to be helpful (i.e. the cluster analysis proposed here), and a battery of psychometric tests. AI is trained on a large database of clinical reports of dyslexic students and on questionnaires and psychometric tests performed by them. The output of BESPECIAL (https://ircai.org/top100/entry/bespecial/) are digital tools (e.g. audiobook, concept maps etc.) and best practices (e.g. presence of tutors, different examination procedures etc.) that are estimated to be more useful for each student. Digital tools will improve the students’ study material and adapting it, using AI, based on students’ needs, whereas the services will be provided to the higher institutions, in order to define standard strategies for inclusive education. In fact, well-known techniques like NLP, OCR, GANs etc. are employed to adapt the material to the specific needs of each student. The above-described AI solution has been conceived within a European project, named VRAIlexia (www.vrailexia.eu), which aims at overcoming all the main difficulties encountered by dyslexic student at university and reducing the gap with respect to non-dyslexic students. In addition to BESPECIAL development, VRAIlexia will lead to: (1) implement a battery of virtual reality test to collect in real-time the skills score of dyslexic students; (2) realize of an online shared repository; (3) create a training path for dyslexia awareness and (4) a memorandum of understanding to spread common inclusion strategies among higher education institutions. Among the several activities provided by VRAIlexia project, the main one consists of providing dyslexic students at university with digital supporting tools that are specific for each of them and, thus, much more effective, in order to decisively reduce the problems they usually encounter and facilitate their academic career. VRAIlexia project aims at changing the perception and developing a model of tools to overcome dyslexic’s main difficulties empowering their motivation and self-esteem. The perspective is to let that Universities can develop strategies for inclusion for fostering all students to discover and point on one’s strengths and values. A further future objective of the Vrailexia project will be to translate the questionnaire into other languages to be administered in other European countries in order to perform a similar clustering approach, permitting to compare the dyslexic profiles based on different languages.
