# Author Correction: Clustering analysis of factors affecting academic career of university students with dyslexia in Italy

**DOI:** 10.1038/s41598-024-53948-7

**Published:** 2024-02-19

**Authors:** Ilaria Benedetti, Marcella Barone, Valentina Panetti, Juri Taborri, Tony Urbani, Andrea Zingoni, Giuseppe Calabrò

**Affiliations:** University of Tuscia, 01100 Viterbo, Italy

Correction to: *Scientific Reports* 10.1038/s41598-022-12985-w, published online 30 May 2022

In the original version of this Article the following authors’ given names and surnames were reversed: Ilaria Benedetti, Marcella Barone, Valentina Panetti, Juri Taborri, Tony Urbani and Andrea Zingoni.

The original Article has been corrected.

